# *α*-Klotho: An Early Risk-Predictive Biomarker for Acute Kidney Injury in Patients with Acute Myocardial Infarction

**DOI:** 10.1155/2023/8244545

**Published:** 2023-03-22

**Authors:** Yuanyuan Pei, Miao Miu, Xue Mao, Wen Chen, Jihong Zhu

**Affiliations:** ^1^Emergency Department, Peking University People's Hospital, Beijing, China; ^2^Emergency Department, Peking University People's Hospital, Qingdao, China

## Abstract

**Background:**

Acute kidney injury (AKI) was a common and serious complication in patients with acute myocardial infarction (AMI). Novel biomarkers and therapies were deficient and imperative for AKI's early diagnosis and therapy after AMI. *α*-Klotho was considered as an early biomarker and potential therapy for AKI recently. Previous studies reported that the expression of *α*-Klotho was decreased in AKI rodents, and supplement of *α*-Klotho alleviated kidney injury. Nevertheless, its effect has not been studied in patients presenting with AMI.

**Methods:**

A total of 155 consecutive diagnosed with AMI at emergency department whose eGFR >60 ml/min *∗* 1.73 m^2^ were enrolled in this prospective observational cohort study which conducted between May 2016 and April 2019 in Peking University People's Hospital. AKI was defined according to the KDIGO criteria in 2012. At admission, the clinical data of patients were collected and serum *α*-Klotho was tested by ELISA. The relationship between *α*-Klotho, serum creatinine, eGFR, systolic pressure, BNP, LVEF, and Hgb of AKI were analyzed and their discrimination performances were compared. The association variables were calculated (adjusted odds ratio) with a confidence interval (CI) of 95% by binary logistic regression. And, we followed up the incidence of CKD and rehospitalization after patients' discharge in one year. Our study was approved by the ethics committee (no. 2016PHB042-01).

**Results:**

AKI incidence was 17.4% (27/155) during hospitalization. Compared to patients without AKI, the AKI group had obviously higher mortality and was more female and had higher incidence of chronic kidney disease, worse cardiac function, more cardiac complications, larger doses of diuretics, and less use of angiotensin-converting enzyme inhibitors/angiotensin receptor blocker. By contrary to previous animal experiments, we found serum *α*-Klotho levels were increased significantly in AKI patients (740.2 ± 306.8 vs. 419.0 ± 272.6 pg/mL, *p* < 0.001). And, the areas under the receiver operating curves indicated serum *α*-Klotho levels had a superior discriminative power for predicting AKI after AMI compared with other risk factors (0.792, 95% CI, 0.706–0.878, *p* < 0.001). Meanwhile, logistic regression model indicates extensive anterior myocardial infarction, Killip classification ≥2 grade, *α*-Klotho ≥516.9 pg/mL, eGFR (decrease per 10 ml/min *∗* 1.73 m^2^), Hgb, and nonuse of ACEI/ARB were the risk factors of AKI after AMI. Moreover, one-year follow-up presented AMI patients developed CKD had higher *α*-Klotho levels (739.7 ± 315.2 vs. 443.8 ± 292.5 pg/mL, *p* = 0.001), but no significant difference in rehospitalization. And, patients with *α*-Klotho ≥516.9 pg/ml was 6.699 times more likely to develop CKD than those with *α*-Klotho <516.9 pg/ml (relative risk 6.699, 95% CI 1.631–27.519, *p* = 0.007).

**Conclusion:**

Compared with traditional cardiac and renal biomarkers, serum *α*-Klotho could be a more appropriate predict biomarker for AKI after AMI in patients' eGFR >60 ml/min *∗* 1.73 m^2^. Higher *α*-Klotho levels are related to the development of AKI during hospitalization and suggest a higher prevalence of CKD after discharge. By contrary to animal experiments, whether the increased expression of *α*-Klotho could be a protective factor secreted by AKI after AMI, is remained to be further studied.

## 1. Introduction

Acute kidney injury (AKI) is well recognized as a common and critical complication in patients with acute myocardial infarction (AMI), which is associated with an increased risk of recurrent myocardial infarction, heart failure, chronic kidney disease (CKD), dialysis, rehospitalization, in-hospital, and long-term mortality. Previous studies have indicated that the incidence of AKI ranged from 7.1 to 29.3% in AMI patients and with poor prognosis [1–3]. Multiple guidelines have emphasized AKI prediction and prevention as a major healthcare priority, for therapeutic options are deficient once AKI occurs [4, 5].

According to 2012 KDIGO criteria of AKI definition [4], serum creatinine and urine output are widely used in the diagnosis of AKI; however, both of them are insensitive. Accordingly, there is an urgent need for better biomarkers for AKI prediction in order to improve the prognosis after AMI. On the other side, searching the novel potential renal protective factors has always been a research hotspot for clinical scientists. Recently, *α*-Klotho has been reported as both a biomarker and replacement therapy for AKI [6, 7]. *α*-Klotho, referred as Klotho, is a 130 kD transmembrane protein with two types: shed and secreted [7, 8]. The extracellular domain of transmembrane Klotho could be cleaved by proteases and released from the kidney into circulation, both as a full-length protein or as Klotho1 and Klotho2 fragments [7, 8]. These circulating forms of Klotho protein are collectively called “soluble” Klotho, playing a biological effect on multiple organs. Presently the research of Klotho mainly focuses on antiaging, antioxidation, regulating bone, calcium, and phosphorus metabolism, inhibiting cell apoptosis, inducing autophagy, and cell senescence [7, 8].

Klotho is confirmed to be mainly expressed in renal distal convoluted tubule and also found in the proximal convoluted tubule, although at lower levels [9]. In various AKI rodent experiments including ischemia-reperfusion injury, cisplatin induced AKI and postrenal unilateral ureteral obstruction models, all indicate a systemic Klotho deficiency state, and applying Klotho supplement possess a considerable potential therapeutic effect [10–12]. Studies in clinical studies also have shown low levels of *α*-Klotho in chronic kidney disease (CKD) and end stage renal disease (ESRD), and low Klotho is reported to be associated with cardiovascular events in hemodialysis patients recently [7, 13, 14]. However, in our previous study, we have reported *α*-Klotho increased significantly in AMI patients without ESRD induced AKI, which with poor early predictive value [15]. Consequently, in order to eliminate the influence of renal dysfunction, we conducted a prospective study in AMI patients with estimated glomerular filtration rate (eGFR) ≥60 ml/min *∗* 1.73 m^2^ to explore the role of *α*-Klotho as AKI develop.

## 2. Methods

### 2.1. Research Subjects

From May 2016 to April 2019, 155 consecutive patients admitted to Peking University People's Hospital in China with a diagnosis with AMI firstly in the emergency department were enrolled in this prospective study. AMI was diagnosed according to the standard criteria [15, 16]. Exclusion criteria were as follows: patients with eGFR <60 ml/min *∗* 1.73 m^2^, septic shock, unable to provide informed consents and who died or were discharged within 48 h. Our study was approved by the hospital ethics committee (No. 2016PHB042-01) and all patients signed informed consent.

### 2.2. Definition

AMI was defined based on a typical chest pain, diagnostic electrocardiographic changes, elevation of troponin I (TNI), and wall motion abnormalities on an ultrasonic cardiogram [16]. AKI was diagnosed according to the criteria of KDIGO criteria, by an increase in serum creatinine of 0.3 mg/dl within 48 h, an elevation of 1.5 fold from the baseline level within the first seven days [4]. eGFR was calculated using modification of diet in renal disease equation for Chinese patients [17]. The AKI stage 1 is defined as an increase in serum creatinine of more than or equal to 0.3 mg/dL or increase to more than or equal to 1.5∼2 fold from baseline. Stage 2 is an increase in serum creatinine to more than 2∼3 fold from baseline. Stage 3 is an increase in serum creatinine to more than 3 fold from baseline, greater than or equal to 4.0 mg/dL, or initiation of renal replacement therapy [4]. The diagnose and development of CKD is defined as abnormalities of kidney structure or function, present for 3 months according to KDIGO 2012 Clinical Practice Guideline [18].

### 2.3. Baseline Characteristics

Clinical data recorded include: age, gender, comorbidities (hypertension and diabetes mellitus), smoking history, heart rate, blood pressure, ST-segment elevation myocardial infarction (STEMI) or non-ST-segment elevation myocardial infarction (USTEMI), cardiac function and complications, contrast volume, laboratory tests, and therapies about AMI. The maximum daily doses of intravenous loop diuretics were counted and converted to furosemide, expressed as 1 mg bumetanide ≈ 20 mg torsemide ≈ 40 mg furosemide. The peak of TNI, creatine kinase-MB (CK-MB), and brain natriuretic peptide (BNP) levels were used in the statistical analysis. All patients were treated with standard anticoagulation and antiplatelet therapies.

### 2.4. Measurement of *α*-Klotho

Once patients were diagnosed with AMI in emergency, serum samples were collected immediately for *α*-Klotho measurements. Serum creatinine was measured, respectively, at admission, third, and seventh day. All samples were centrifuged at 1500 rpm for 10 minutes and then stored at −80°C until detection. All biomarkers were measured in duplicate by a single enzyme-linked immunosorbent assay (ELISA) according to the instruction of manufacturer. *α*-Klotho ELISA kit was purchased from R & D Systems (DY5334-05).

### 2.5. One-Year Followup

After patients' discharge, we carried on a one-year followup to observe CKD's appearance and rehospitalization rate through telephone enquiry. The reason for readmission included unstable angina, recurrent myocardial infarction and heart failure.

### 2.6. Statistical Analysis

All variables were tested for normal distribution by the Kolmogorov–Smirnov test and the descriptive statistics were summarized and displayed as the mean ± standard deviation or the median (25∼75%). Continuous variables and normal distribution data were compared using independent sample *t*-tests. Categorical data were tested by the Chi-square test or Fisher's exact test. During hospitalization, all data were compared between the AKI group and non-AKI group. Binary logistic regression was generated using the Enter mode, and the association variables were calculated (adjusted odds ratio) with a confidence interval (CI) of 95%. For the followup, we compared the *α*-Klotho levels of CKD group with those of non-CKD group, and the rehospitalization group with nonrehospitalization group. A *p* value <0.05 was defined statistical significantly. Discrimination was assessed based on the area under a receiver operating characteristic curve (AUROC). And, the AUROC analysis was conducted to calculate the cut-off values, sensitivity, and specificity, and the cut-off points were estimated by determining the best Youden index. All analyses were performed by SPSS 23.0 software.

## 3. Results

### 3.1. Baseline Characteristics

155 patients were enrolled totally, 135 men and 20 women participated respectively in this study. Among them, 100 patients presented STEMI and 55 patients had USTEMI. The demographic, clinical presentation, laboratory tests and therapies were listed in Table 1. Compared with the non-AKI group, patients with AKI contained more women and with higher incidence of CKD. In cardiac manifestation, the AKI group had lower systolic pressure, faster heart rate, higher Killip class, more STEMI, more extensive anterior myocardial infarction, severer cardiac function, more cardiac complications including different types of arrhythmias and acute heart failure. In terms of laboratory tests, we found that it was consistent in renal function of both groups at admission, but during hospitalization AKI group developed a deterioration in renal function. Moreover, AKI group's myocardial necrosis was more serious, tending to TNI and CK-MB were obviously higher. And larger doses of diuretics, less use of angiotensin-converting enzyme inhibitors/angiotensin receptor blocker (ACEI/ARB), more device-assisted treatment were found in AKI groups.

### 3.2. Incidence of AKI

In this study, 17.4% (27/155) patients developed AKI in the first seven days according to the KDIGO definition. In AKI groups, 25 patients were at AKI 1 stage overwhelmingly, and only 1 patient was at AKI stage 2 and 3 separately.

### 3.3. Discrimination Performance and Accuracy of Klotho for AKI after AMI

**Figure 1:**
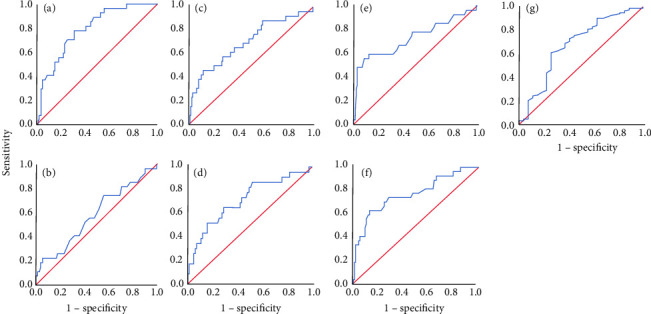
Comparison of discrimination performance of Klotho, SCr, eGFR, BNP at admission and systolic pressure, LVEF, and hemoglobin regarding the incidence of AKI after AMI. The respective AUROC are 0.792 (*p* < 0.001), 0.583 (*p*=0.179), 0.698 (*p*=0.001), 0.717 (*p*=0.001), 0.726 (*p* < 0.001), 0.765 (*p* < 0.001), and 0.680 (*p*=0.003) according to (a)–(g). (a) *α*-Klotho at admission. (b) SCr at admission. (c) eGFR at admission. (d) BNP at admission. (e) Systolic pressure. (f) LVEF. (g) Hemoglobin.

As shown in Table 2, Klotho increased significantly in the AKI group at admission. The ROC curves of *α*-Klotho, serum creatinine, eGFR, BNP at admission and systolic pressure, LVEF, and hemoglobin in predicting the development of AKI after AMI were displayed in Table 3 and Figure 1. Areas indicated that serum levels of *α*-Klotho had modest discriminative powers in prediction of AKI than creatinine (AUROC 0.792, 95% confidence interval (CI), 0.706 to 0.878, *p* < 0.001 and AUROC 0.583, 95% CI, 0.460 to 0.706, *p*=0.179). The sensitivity and specificity were 0.778 and 0.694 for *α*-Klotho, and its cut-off value was 516.9 pg/ml.

### 3.4. Risk Factors of AKI after AMI

We created a regression model to elucidate the risk factors associated with AKI in AMI patients. In the final regression model, the following variables were included: extensive anterior myocardial infarction, Killip classification ≥2 grade, eGFR, LVEF, TNI, Hgb, *α*-Klotho, and nonuse of ACEI/ARB. The major risk factors for AKI were extensive anterior myocardial infarction (odds ratio [OR] = 7.525, 95% CI 1.039–54.487, *p*=0.046), Killip classification ≥2 grade (OR = 7.797, 95% CI 1.649–36.878, *p*=0.01), *α*-Klotho (≥516.9 pg/ml) (OR = 7.357, 95% CI 1.516–35.702, *p*=0.013), eGFR (decrease per 10 ml/min *∗* 1.73 m^2^) (OR = 1.048, 95% CI 1.006–1.091, *p*=0.023), Hgb (OR = 1.052, 95% CI 1.005–1.102, *p*=0.030), and nonuse of ACEI/ARB (OR = 5.401, 95% CI 1.148–25.414, *p*=0.033) (Table 4).

### 3.5. Length of Hospital Stay and Mortality

Compared with the non-AKI group, the AKI group had significantly longer hospitalization [14 (12.20) *d* vs. 9 (7.14) *d*, *p* < 0.001] and higher mortality (22.2% vs. 0.0%, *p* < 0.001). 6 patients developing AKI died in the hospital and all patients without AKI were discharged smoothly.

### 3.6. Followup Data

We followed up the discharged patients for one year, mainly counting the rehospitalization rate of patients due to recurrent myocardial infarction and heart failure, as well as the incidence of CKD. In the AKI group, 6 patients died in hospital, 2 patients were lost to followup, 6 patients developed CKD (6/19, 31.6%), and 9 (9/19, 47.4%) were rehospitalized within one year due to the above-given cardiac diseases. In the non-AKI group, 13 patients were lost to followup, and in the remaining 115 patients, 4 patients with CKD (3.5%) and 7 patients (6.1%) were rehospitalized within one year.

The occurrence/development of CKD and the rehospitalization rate were observed during followup, whereafter the baseline *α*-Klotho levels were compared. It was found that patients with higher *α*-Klotho levels developed more CKDs [739.7 ± 315.2 (*n* = 10) vs. 443.8 ± 292.5 pg/ml (*n* = 124), *p*=0.001] but had no statistical difference in the rehospitalization rate [524.4 ± 329.9 (*n* = 16) vs. 458.5 ± 300.3 pg/ml (*n* = 118), *p*=0.405]. Ulteriorly, we calculated the relative risk (RR) of these variables, and we found that patients with *α*-Klotho ≥516.9 pg/ml was 6.699 times more likely to develop CKD than patients with *α*-Klotho <516.9 pg/ml (RR 6.699, 95% CI 1.631–27.519, *p*=0.007). Meanwhile, *α*-Klotho ≥516.9 pg/ml was 1.538 times more likely to rehospitalization than those with *α*-Klotho <516.9 pg/ml (RR 1.538, 95% CI 0.516–4.580, *p*=0.437).

## 4. Discussion

In this study, we aim to examine *α*-Klotho's early diagnostic value in diagnosis of AKI after AMI, as well as to predict effect of CKD and readmission. The main findings of our study can be summarized as follows: (1) *α*-Klotho is a superior biomarker than serum creatinine for AKI in AMI patients with eGFR ≥60 ml/min *∗* 1.73 m^2^, (2) Higher *α*-Klotho levels at admission indicates greater morbidity of CKD, and (3) with numerous studies finding *α*-Klotho decreased in AKI rodent models, our team firstly reported that *α*-Klotho increased significantly in AMI induced AKI patients, which deserves further research.

Development of AKI following AMI is regarded as an important complication in patients with AMI due to its enhancive in-hospital and long-term mortality and subsequent risk of CKD/ESRD. Previous observational studies showed the related risk factors of AKI after AMI, including use of hypertension, diabetes, CKD, hemodynamic instability, cardiogenic shock, decreased cardiac output, contrasts, and medicines, which was mostly consistent with the clinical data of our study [1, 2, 19–21]. In this study, the incidence of AKI was 17.4%, and an approximate quarter of patients with AKI died in hospital. It worth noting that even we enrolled patients with eGFR ≥60 ml/min *∗* 1.73 m^2^, there still has an increased prevalence of CKD in AKI group. Furthermore, AKI group tend to have more female, cardiac dysfunction and complications, less use of ACEI/ARB, more use of loop diuretics, and supportive therapies. And, logistic regression model indicates extensive anterior myocardial infarction, Killip classification ≥2 grade, *α*-Klotho ≥516.9 pg/mL, eGFR (decrease per 10 ml/min *∗* 1.73 m^2^), Hgb, and nonuse of ACEI/ARB are the risk factors of AKI after AMI.

According to AKI's definition in 2012, it takes at least 48 hours to observe the dynamic variation of serum creatinine for AKI's diagnosis. For the acknowledgement of early detection of AKI may allow better therapy and potentially avoid its undesirable prognosis, we compared the precision and discriminative ability of serum creatinine and *α*-Klotho for AKI prediction after AMI at admission. The results indicated that *α*-Klotho may be a more sensitive and reliable biomarker for AKI. Compared with serum creatinine, *α*-Klotho was significantly elevated in the AKI group of AMI on admission, when AKI had not developed. And, at this moment, there was no statistical difference in serum creatinine between the two groups. In addition, the comparison of other conventional risk factors included hemoglobin, blood pressure, eGFR, BNP, and LVEF of the predictive value for AKI after AMI were conducted. We found that, compared with traditional renal and other indicators, the factors represented cardiac function (SBP, LVEF, and BNP) might have better predictive value, with AUROC greater than 0.7. However, it was still not as good as *α*-Klotho. The cut-off value of *α*-Klotho was 516.9 pg/mL (sensitivity, 0.778; specificity, 0.694; Youden index, 0.472); and AUROC was 0.792.

Emerging evidence has revealed that *α*-Klotho deficiency may be an early event in AKI rodent models, and a pathogenic factor that exacerbates acute kidney damage and contributes to long-term consequences [6–9]. Studies in human and animal models have shown low levels of serum *α*-Klotho in CKD and ESRD, and treatment with *α*-Klotho could improve kidney damage in rodent models [6, 7]. Currently, few studies have been focused on *α*-Klotho expressions in human AKIs. Seibert et al. [22] have reported that serum *α*-Klotho was increased in AKI patients and progressively decreased in patients with CKD stage 1 to 5, and in this study, the etiology of AKI includes infection, parenchymal renal disease, acute tubular necrosis, prerenal kidney failure, cardiorenal syndrome (CRS), and other renal disease. Another research on renal *α*-Klotho expression determined by immunohistochemical staining showed that renal *α*-Klotho expression decreased significantly according to the severity of AKI, and that low expression was associated with a poor short-term outcome [23], which suggests that Klotho expression is opposite in circulation and kidney when AKI happens, manifested as high circulating Klotho and low organ Klotho expression.

AMI induced AKI belongs to type I CRS. Jerin et al. [24] found that *α*-Klotho levels of AKI group increased in few hours after cardiac surgery and decreased in 48 hours postoperatively. Qian et al. [25] discovered higher urine Klotho levels in AKI groups after cardiac surgery, which was associated with poor short-term renal prognosis. These results were consistent with our study on AMI related AKI.

The possible reasons for the opposite results in animal and human researches could be that animal models of AKI were established by drugs or ischemia-reperfusion injury, resulting in recoverable kidney damage, while human AKIs might be relieved properly. And, there were various mechanisms of animal AKI models, which might explain the reason for the opposite clinical findings in the present study. Therefore, we speculate that the elevated level of *α*-Klotho might be a negative feedback phenomenon of the body, suggesting that *α*-Klotho might be a protective factor indirectly. And, in an AMI rodent model, *α*-Klotho was found to inhibit the expression of proinflammatory cytokines in the peri-infarct regions and significantly attenuate the apoptosis and production of intracellular reactive oxygen species by myocardial ischemia/reperfusion injury [26]. Further studies are needed on whether Klotho could be considered as a self-protection biomarker during AKI after AMI. Dynamic changes of *α*-Klotho profiles might be conducive to the potential hypothesis.

The study also has its limitations. Firstly, the enrolled sample capacity was relatively small, and all patients were from a single center. Secondly, *α*-Klotho was only detected on admission, although it is more predictable for AKI, but we did not monitor the creatinine and *α*-Klotho to observe continuous changes. Thirdly, the biomarker needs to be proven in a general population without exclusion criteria, including who died in 48 h since it could not diagnose AKI according to current definition. Since Klotho is definitely declined in chronic renal failure patients, we excluded this part of the population in the study design stage and it is necessary to explore the dynamic evolutions of Klotho levels in AMI patients complicated with CKD 4-5 stages when AKI occurs.

In summary, the study provides sound evidence that Klotho could be a better biomarker for AKI in AMI patients with eGFR >60 ml/min *∗* 1.73 m^2^. Elevated *α*-Klotho levels are related to the development of AKI during hospitalization and suggest a higher prevalence of CKD after discharge. On contrary to rodent experiments, whether the increased expression of Klotho is a self-protective factor secreted by AKI after AMI, still remains further study.

## Figures and Tables

**Table 1:** Baseline characteristics in AMI patients with or without AKI.

Variables	Total (*n* = 155)	AKI group (*n* = 27)	Non-AKI group (*n* = 128)	*p* value
*Demography*				
Age (years)	61 ± 12	65 ± 14	61 ± 12	0.457
Female (%)	20(12.9)	9 (33.3)	11 (8.6)	0.002
Hypertension (%)	83 (53.5)	15 (55.6)	68 (53.1)	0.818
DM (%)	84 (29.5)	7 (25.9)	37 (28.9)	0.755
Prior MI (%)	22 (14.2)	4 (14.8)	18 (14.1)	0.561
CHF (%)	3 (1.9)	1 (3.7)	2 (1.6)	0.439
CKD (%)	8 (5.2)	7 (25.9)	1 (0.8)	<0.001
Cerebral infarction (%)	22 (14.2)	7 (25.9)	15 (11.7)	0.069
Previous PCI (%)	19 (12.3)	3 (11.1)	16 (12.5)	0.570
Previous CABG (%)	1 (0.1)	0 (0.0)	1 (0.8)	0.826
Chronic lung disease (%)	4 (2.6)	2 (7.4)	2 (1.6)	0.140
Dislipidemia (%)	26 (16.8)	7 (25.9)	19 (14.8)	0.166
Smoking history (%)	108 (69.7)	16 (59.3)	92 (71.9)	0.195

*Clinical presentation*				
Systolic pressure (mmHg)	121 ± 25	104 ± 31	124 ± 22	0.014
Diastolic pressure (mmHg)	72 ± 16	62 ± 16	74 ± 15	0.079
Heart rate (bpm)	76 (68, 90)	96(83, 120)	72(66, 84)	<0.001
STEMI (%)	100 (64.5)	23 (85.2)	77 (60.2)	0.014
Extensive anterior myocardial infarction (%)	29 (18.7)	15 (55.6)	14 (10.9)	<0.001
Killip grade ≥III stage (%)	15 (9.7)	12 (52.2)	3 (3.6)	<0.001
Cardiogenic shock (%)	10 (6.5)	9 (33.3)	1 (0.8)	<0.001
Ventricular tachycardia or fibrillation (%)	9 (5.8)	7 (25.9)	2 (1.6)	<0.001
Atrial fibrillation (%)	11 (7.1)	7 (25.9)	4 (3.2)	<0.001
Atrioventricular block (%)	1 (0.6)	0 (0.0)	1 (0.8)	0.826
Acute heart failure (%)	13 (8.4)	11 (40.7)	2 (1.6)	<0.001

*Laboratory tests*				
Hemoglobin (g/L)	137 ± 20	126 ± 21	137 ± 19	0.007
WBC (×10^9^/L)	8.8 (6.9, 11.3)	11.4 (8.8, 15.0)	8.6 (6.8, 10.5)	0.002
PLT (×10^9^/L)	196 ± 66	202 ± 77	208 ± 63	0.677
SCr at admission (*μ*mol/L)	73.0 ± 15.8	78.2 ± 20.4	73.3 ± 14.6	0.140
SCr Max (*μ*mol/L)	79.0 (67.0, 91.0)	116.0 (103.0, 155.0)	73.5 (64.3, 85.0)	<0.001
eGFR at admission (ml/min^*∗*^1.73 m^2^)	92.77 (83.36, 100.46)	84.70 (64.20, 95.49)	93.99 (85.05, 101.22)	0.001
The valley eGFR (ml/min^*∗*^1.73 m^2^)	90.06(72.16, 99.35)	48.21 (40.68, 67.38)	93.80 (84.93, 101.22)	<0.001
BUN (mmol/l)	5.55 (4.25, 7.06)	7.69 (6.61, 10.20)	5.11 (4.11, 6.50)	<0.001
TNI Max (ng/ml)	10.5 (2.7, 55.0)	78.0 (15.2, 81.0)	7.8 (1.8, 30.9)	<0.001
CK-MB (U/L)	39.9 (10.2, 190.0)	228.3 (32.7, 300.0)	33.9(7.9, 123.0)	<0.001
BNP at admission (ng/ml)	148.5 (75.4, 288.8)	300.0 (132.0, 977.0)	124.5 (63.0, 250.5)	0.001
BNP Max (ng/ml)	167.0(79.7, 466.3)	783.8 (300.0, 1599.0)	131 (63.0, 270.0)	<0.001
FBG (mmol/L)	6.35 (5.19, 8.40)	7.55 (5.99, 10.68)	6.20 (5.09, 8.11)	0.010
Albumin (g/L)	38.4 ± 3.8	36.5 ± 4.0	38.8 ± 3.7	0.005
CHO (mmol/L)	4.37 ± 1.08	4.44 ± 0.88	4.36 ± 0.11	0.713
TG (mmol/L)	1.51 (1.06, 2.07)	1.27 (0.99, 2.13)	1.55 (1.08, 2.07)	0.295
LDL (mmol/L)	2.74 ± 0.87	2.63 ± 0.79	2.76 ± 0.88	0.507
HDL (mmol/L)	0.95 (0.83, 1.09)	1.03 (0.86, 1.26)	0.94 (0.82, 1.06)	0.066
LVEF (%)	60 ± 11	52 (38, 63)	64 (58, 68)	<0.001

*Therapies*				
Use of furosemide (mg/d)	0 (0, 20)	40 (40, 80)	0 (0, 10)	<0.001
Intravenous isosorbide dinitrate (%)	104 (67.1)	19 (70.4)	85 (66.4)	0.823
Nonuse of ACEI/ARB (%)	54 (34.8)	18 (66.7)	36 (28.1)	<0.001
Use of *β*-blockers (%)	130 (83.9)	22 (82.5)	108 (84.4)	0.710
Use of statins (%)	154 (99.4)	26 (96.3)	128 (100.0)	0.221
Vasoactive medication (%)	29 (18.7)	17 (63.0)	12 (9.4)	<0.001
IABP (%)	12 (7.7)	7 (25.9)	5 (3.9)	0.037
In-hospital PCI (%)	111 (71.6)	18 (66.7)	93 (72.7)	0.530
In-hospital CABG (%)	10 (6.5)	2 (7.4)	8 (6.3)	0.686
In-hospital mechanical ventilation (%)	10 (6.5)	5 (18.5)	5 (3.9)	0.005
Contrast volume (ml)	200 (100, 228)	200 (100, 300)	200 (100, 225)	0.480
Thrombolysis therapy (%)	4 (2.6)	1 (3.7)	3 (3.6)	0.685

Data expressed as mean ± standard deviation, *n* (%), or median (interquartile range). DM, diabetes mellitus; MI, myocardial infarction; CHF, congestive heart failure; CKD, chronic kidney disease; PCI, percutaneous coronary intervention. CABG, coronary artery bypass grafting; STEMI, ST-segment elevation myocardial infarction; SCr, serum creatine; eGFR, estimated glomerular filtration rate; FBG, fasting blood glucose; LVEF, left ventricular ejection fraction; Vasoactive medication included dopamine, dobutamine, and norepinephrine. Compared with non-AKI group, *P* < 0.05 was considered significantly.

**Table 2:** Serum *α*-Klotho protein levels in AMI patients.

	Total (*n* = 155)	AKI group (*n* = 27)	Non-AKI group(*n* = 128)	*p* value
*α*-Klotho (pg/ml)	518.0 ± 328.5	742.2 ± 497.4	470.3 ± 257.2	<0.001

Compared with non-AKI group, *P* < 0.05 was considered significantly.

**Table 3:** The areas under the receiver operating characteristic curves for serum levels of *α*-Klotho and other conventional risk factors to predict AKI after AMI.

	AUROC	95% CI	*p* value	Cut-off value	Sensitivity	Specificity
SCr at admission (*μ*mol/L)	0.583	0.460–0.706	0.179	70.5	0.741	0.444
eGFR at admission (ml/min*∗*1.73 m^2^)	0.698	0.579–0.817	0.001	75.75	0.462	0.883
SBP (mmHg)	0.726	0.601–0.851	<0.001	100	0.556	0.914
Hgb (g/L)	0.680	0.559–0.801	0.003	136	0.741	0.614
LVEF (%)	0.765	0.657–0.873	<0.001	54.4	0.630	0.866
BNP at admission (ng/ml)	0.717	0.593–0.842	0.001	297.8	0.522	0.843
Klotho at admission (pg/ml)	0.792	0.706–0.878	<0.001	516.9	0.778	0.694

AUROC, area under the receiver operating characteristic curves; CI, confidence interval.

**Table 4:** The binary logistic regression model for predicting AKI in AMI patients.

Correlates	OR	95% CI	*p* value
Extensive anterior myocardial infarction	7.525	1.039–54.487	0.046
Killip classification ≥2 grade	7.797	1.649–36.878	0.010
Klotho (≥516.9 pg/ml)	7.357	1.516–35.702	0.013
eGFR (decrease per 10 ml/min *∗* 1.73 m^2^)	1.048	1.006–1.091	0.023
TNI (ng/ml)	0.973	0.950–0.995	0.018
Nonuse of ACEI/ARB	5.401	1.148–25.414	0.033
LVEF (%)	1.023	0.947–1.106	0.564
Hgb (g/L)	1.052	1.005–1.102	0.030

## Data Availability

The datasets used or analyzed about the study could be available from the corresponding author on reasonable request.
